# Early Detection of Motor Dysfunction in the SOD1^G93A^ Mouse Model of Amyotrophic Lateral Sclerosis (ALS) Using Home Cage Running Wheels

**DOI:** 10.1371/journal.pone.0107918

**Published:** 2014-09-30

**Authors:** Ellen J. Bennett, Richard J. Mead, Mimoun Azzouz, Pamela J. Shaw, Andrew J. Grierson

**Affiliations:** Sheffield Institute for Translational Neuroscience, Department of Neuroscience, University of Sheffield, Sheffield, United Kingdom; University of Edinburgh, United Kingdom

## Abstract

The SOD1^G93A^ mouse has been used since 1994 for preclinical testing in amyotrophic lateral sclerosis (ALS). Despite recent genetic advances in our understanding of ALS, transgenic mice expressing mutant SOD1 remain the best available, and most widely used, vertebrate model of the disease. We previously described an optimised and rapid approach for preclinical studies in the SOD1^G93A^ mouse. Here we describe improvements to this approach using home cage running wheels to obtain daily measurements of motor function, with minimal intervention. We show that home cage running wheels detect reductions in motor function at a similar time to the rotarod test, and that the data obtained are less variable allowing the use of smaller groups of animals to obtain satisfactory results. This approach refines use of the SOD1^G93A^ model, and reduces the number of animals undergoing procedures of substantial severity, two central principles of the 3Rs (replacement, reduction and refinement of animal use in research). The small group sizes and rapid timescales enable affordable large-scale therapeutic pre-screening in the SOD1^G93A^ mouse, as well as rapid validation of published positive effects in a second laboratory, one of the major stumbling blocks in ALS preclinical therapy development.

## Introduction

ALS is the most common adult onset neurodegenerative disease affecting motor neurons. It is uniformly fatal, and there is only one approved pharmacological treatment, Riluzole, which at best extends survival by an average of only 3–4 months [1], [2]. ALS is a multi-factorial disease, and new treatments that target a range of pathways are in preclinical development [3].

The standard in vivo model used for preclinical and proof of concept studies in ALS is a transgenic mouse overexpressing a human superoxide dismutase 1 minigene bearing a pathogenic glycine to alanine mutation at amino acid position 93 (the SOD1^G93A^ mouse) [4]. It is clear that this is pathophysiologically a valid model of ALS (since SOD1 mutations cause the disease in humans), and it is known that the model has predictive validity (since Riluzole, the only drug approved for human ALS patients produces an increase in survival in SOD1^G93A^ mice [5]). However, despite these advantages, it is clear that the model is not perfect as there have been a number of studies reporting beneficial effects of drugs in SOD1^G93A^ mice that have subsequently resulted in failure in human trials. This problem was highlighted by a meta-analysis that revealed a bias in the publication of positive preclinical results, and significant shortcomings in experimental design in some of the studies [6]. However multiple laboratories have generated mutant SOD1 mice bearing different mutations, and the overt phenotype of motor axonopathy leading to motor neuron death and astrogliosis are very similar in each of these models, and compare well with familial and sporadic forms of human ALS [7]–[9].

In recent years there has been an increase in translational research conducted in academic settings. One reason for academic involvement is the perception that Industry-led translational medicine may be failing. This is because about 90% of drugs that look promising in preclinical studies currently do not reach the marketplace. The major reasons for this are lack of efficacy; toxicity; and safety concerns [10]. In fact for central nervous system (CNS) disorders the statistics are worse than this – only about 1 in 20 drugs emerging from pre-clinical evaluation will succeed in human trials.

As the number of animals being used for research continues to rise, this must be weighed against the requirement to implement the principles of the 3Rs (replacement, reduction and refinement of animal use in research). For ALS mouse models, perhaps the biggest opportunities are for refinement and reduction, since progressive paralysis in end stage mice causes severe distress, and large cohort sizes are required to obtain statistically valid results. Previously, we proposed that earlier endpoints could be used during early stage preclinical investigations in vivo, and described a methodology that refined use of the SOD1^G93A^ mouse model for pre-clinical studies [11]. This approach resonates with several studies that report a similar early reduction in motor performance [12], [13], and correlates with a loss of functional motor units as measured by electromyography [14] and the onset of denervation of motor end-plates [15]–[17]. Here, we determine the efficacy of home cage running wheels as an alternative early readout of motor dysfunction in the SOD1^G93A^ mouse model, and compare this to current use of the rotarod in the same model. We show that home cage running wheels can be used to detect motor dysfunction at an early stage, and that running wheel data is inherently less variable than that obtained using rotarod, thereby allowing refinement and reduction in early-stage preclinical testing.

## Methods

### Ethics Statement

All experiments were conducted according to the Animal (Scientific Procedures) Act 1986, under Project License 40/3089 reviewed and approved by the University of Sheffield Ethical Review Sub-Committee, and the UK Animal Procedures Committee (London, UK). The UK Home Office code of practice for the housing and care of animals used in scientific procedures was followed. We adhere to the ARRIVE guidelines for reporting animal research [18].

### Transgenic C57BL/6 SOD1^G93A^ model of ALS

As previously described [11], we use a congenic strain of transgenic C57BL/6 SOD1^G93A^ mice which were originally obtained from the Jackson Laboratory [4], and hemizygous transgenic males were backcrossed to C57BL/6 females (Harlan UK, C57BL/6 J OlaHsd substrain) for >20 generations. Transgenic SOD1^G93A^ mice were identified by ear biopsy and PCR as previously described [11]. Transgene copy number was routinely determined using quantitative PCR [11]. The humane endpoint used in our experiments is loss of self-righting ability within 10 seconds of being placed on the back, or weight loss of >30% for 72 hours. Mice were provided with wet mashed food in their cages at the first sign of hind limb paralysis. At this point the cage bedding material was changed to paper towel to reduce dust and allow for easier locomotion.

### Housing

Mice were bred in a specified pathogen free (SPF) environment, and transferred to a conventional facility for the studies described here. The facility uses a 12 h light/dark cycle, and a standardised room temperature of 21°C. Mice were fed 2018 rodent diet (Harlan, UK) *ad libitum*. Cages were lined with fine sawdust (eco-pure chips premium). Paper wool (Datesand, UK) was used as bedding material, and plastic houses were provided in each cage.

### Study Design

We followed principles of experimental design in line with published recommendations [19]. Based on our previous studies, we required groups of at least 14 mice to achieve appropriate power for rotarod analysis, and up to 15 mice per group for survival analysis [11]. Since one purpose of this study was a direct comparison with existing methods, we chose to use groups of 15 mice. Previous studies have demonstrated that wild type female C57BL/6 mice show a greater propensity for spontaneous home cage running wheel activity [20], therefore we used single-sex experimental cohorts of female mice. It should be noted that on the C57BL/6 background we find no differences in disease onset, progression or lifespan in male and female mice [11].

**Figure 1:**
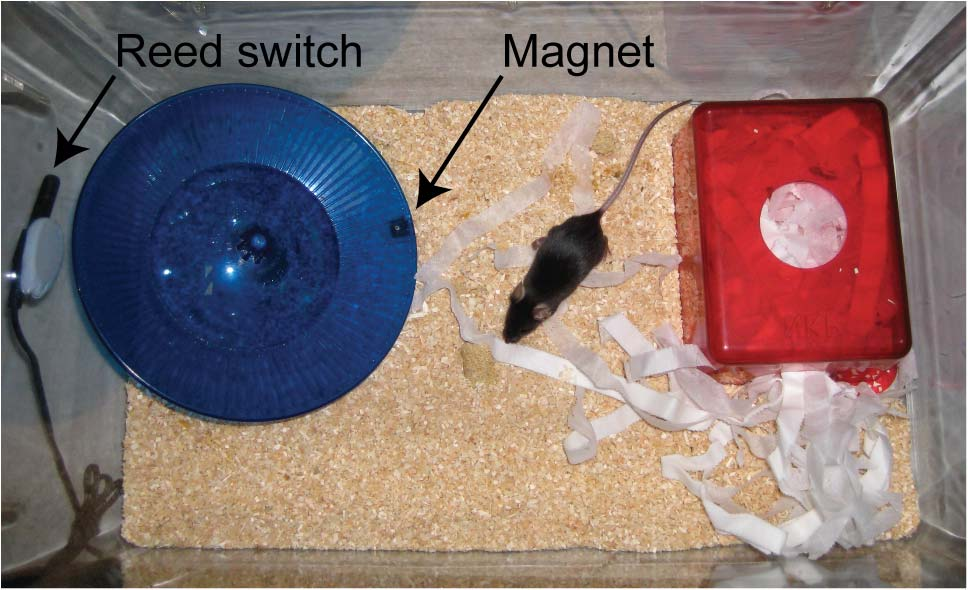
A typical cage set-up for assessment of home cage running activity. The angled Fast Trac running wheel is located such that the magnet is sufficiently close to the reed switch positioned on the exterior of the cage.

Between 26 and 32 days of age, litter-matched SOD1^G93A^ mice were randomly assigned to one of two experimental groups. The first group were housed individually in 36×21×18.5 cm (length × width × height) cages with a Fast Trac running wheel (LBS Biotech, UK) 37.8 cm circumference, fitted on the underside with a 5×10 mm neodymium magnet (First4magnets.com), and mounted at a height of 4 cm on a lubricated axle at an angle of 25° below horizontal. Almost identical wheels are also available in the USA e.g. Inno Wheel (Bio-Serv, USA). The wheel was positioned so that it is at a distance of 5–10 mm from the sides of the cage. Voluntary running wheel activity was monitored using a reed switch coupled to a bicycle odometer (Cateye Velo series) (Figure 1). This experimental set-up is based on that previously described for experiments investigating the effects of exercise training in mice [20]. The second group were housed in identical cages, but with a Fast Trac wheel that did not rotate.

Additional preclinical studies were performed as described in Table 1. Data obtained from placebo cohorts were analysed to determine the reliability of running wheel data between experiments. Group sizes vary depending on whether the studies were powered for survival or running wheel endpoints.

**Table 1:** Details of the experimental cohorts reported in this paper.

				Distance		Time		Average speed	
Study	Start date	N	Vehicle^1^	Mean age of 20% reduction ±(SD)	CV (%)	Mean age of 20% reduction ±(SD)	CV (%)	Mean age of 20% reduction ±(SD)	CV (%)
**1**	01/07/2009	15	-	44.27±3.31	7.4	45.57±4.54	10	47.13±3.76	8.0
**2**	21/11/2009	8	A	41.75±3.28	7.9	43.13±3.98	9.2	43.38±4.34	10.0
**3**	28/04/2010	9	B	43.22±4.44	10.3	44.56±3.40	7.6	48.25±5.8	12.0
**4**	28/10/2010	7	B	44.71±5.44	12.2	44.29±9.62	21.7	45.80±1.92	4.2
**5**	12/01/2011	6	B	44.67±4.23	9.5	45.00±3.69	8.2	46.00±5.10	11.1
**6**	06/05/2011	12	C	45.21±3.40	7.5	46.07±3.79	8.2	46.42±2.31	5.0
**7**	31/05/2011	14	B	42.86±4.09	9.5	43.93±5.02	11.4	48.67±4.52	9.3
**All**		**71**		**43.85±3.93**	**9.0**	**44.77±4.84**	**10.8**	**46.77±4.21**	**9.0**

Results from the initial cohort (study 1) are shown alongside 6 subsequent placebo cohorts (studies 2–7).

1 Vehicle treatments were as follows: A, in chow (continuously); B, in water (continuously); C, intraperitoneal (daily 5 ml/kg).

CV  =  coefficient of variation.

### Behavioural Measures

Wheel running activity was recorded every morning, and subsequently adjusted for the circumference of the running wheel. Distance (km) was recorded as the total number of wheel revolutions multiplied by the circumference. Time (hours) was recorded as continuous wheel activity, with a maximum gap of 5 s between rotations. Average speed (km/h) is calculated as the total distance divided by the time for a 24 h period. Maximum speed is the highest recorded running speed in a 24 h period. Because the bedding material was often found to be wrapped around the wheel support, Fast Trac wheels and odometers were checked every morning to ensure the wheels were freely rotating, and the reed switches were functional. Very occasionally data points were censored because of jammed wheels caused by bedding material, an odometer that failed to reset, a detached reed switch, or a water bottle leak. Across all studies presented here we censored on average just 0.23 days per mouse.

A Ugo Basile 7650 accelerating rotarod (set to accelerate from 4–40 rpm over 300 seconds) was used to measure motor function. Rotarod training was performed over 3 consecutive days, with two trials per day. Subsequently this test was performed at weekly intervals during the afternoon. On each occasion the mice were tested twice, with a rest period between runs. The best performance, measured as latency to fall in seconds, was used for analysis. The minimum threshold for recording rotarod activity was 5 seconds. For analysis of disease onset and progression we calculated the time at which individual mice reached a 20% decline in rotarod performance, compared to their specific post-training baseline.

Body weight was determined at weekly intervals until 120 days of age, at which point SOD1^G93A^ mice were weighed daily to monitor mice for signs of severe disease-associated weight loss. Mice were also scored for tremor, hind-limb splay and overall neurological deficit using a previously reported scoring system [11]. Disease onset was defined as the point at which both enhanced tremor and defective hind-limb splay were first observed. From 125 days of age SOD1^G93A^ mice were monitored twice daily using a distress scoring system [11], until they reached the humane endpoint for euthanasia (see above). At this point mice were euthanized with an overdose of anaesthetic (intraperitoneal injection of 20 ml/kg pentobarbitone).

### Statistics

Prism 6.0 (GraphPad Software Inc) was used for statistical analysis. Details of specific tests are stated in the text. G*Power 3.1 was used for all power calculations [21].

## Results

### Validation of home cage running wheels in female C57BL/6 J OlaHsd mice

**Figure 2:**
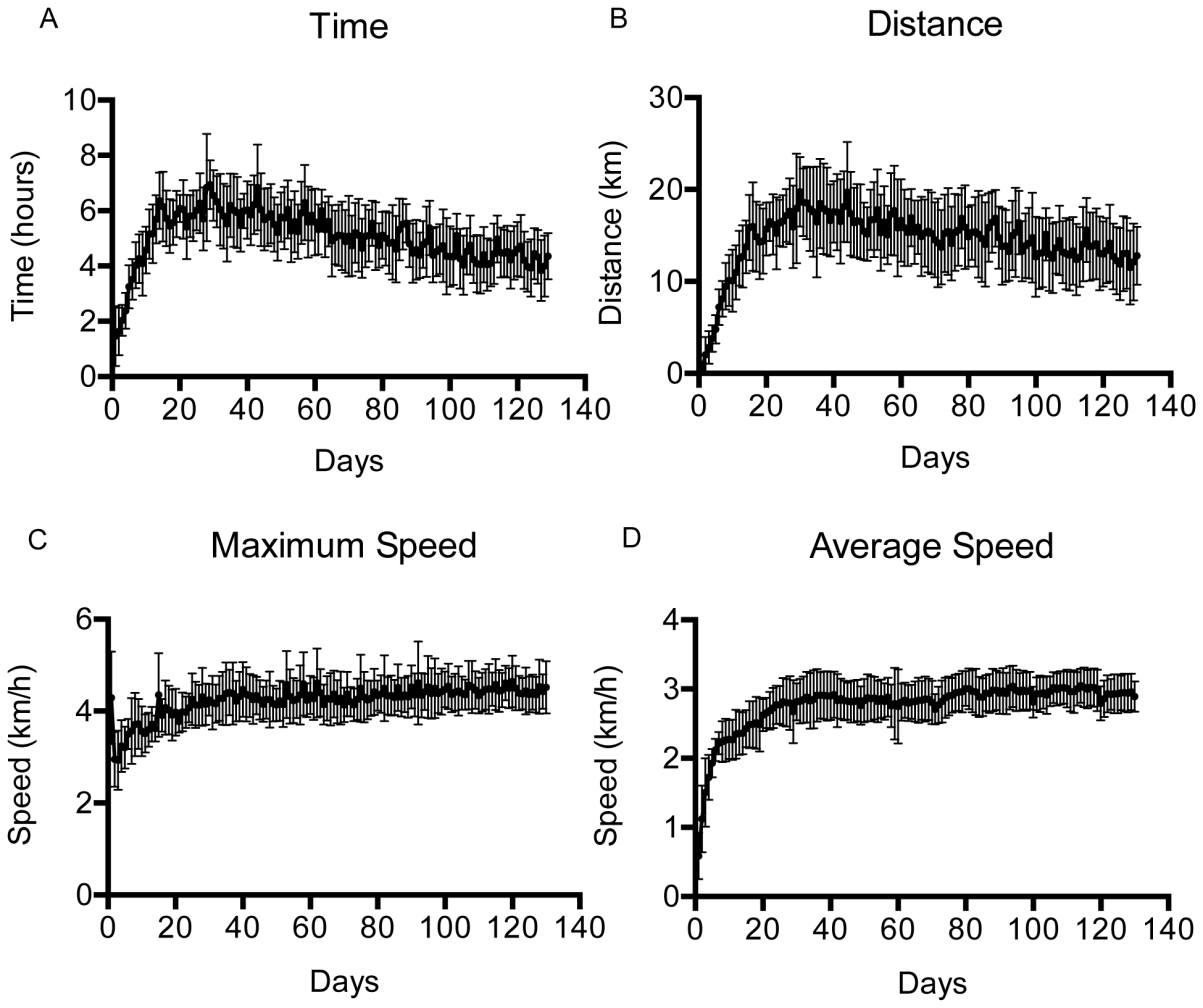
Running wheel activity of wild type C57BL/6 J OlaHsd mice. Mean (± SD) time (A), distance (B), maximum speed (C) and average speed (D) were recorded for 130 days in a group of 15 mice, starting at 6 weeks of age.

We first investigated Fast Trac running wheel activity in a cohort of 15 C57BL/6 J OlaHsd female mice, beginning at 6 weeks of age. These are the same C57BL/6 substrain as our SOD1^G93A^ mouse colony [11]. The typical home cage set-up is shown in Figure 1. Data for running time, distance, maximum speed and average speed were recorded daily for 130 days (Figure 2A–D). As previously reported, the mice showed an increase in activity over the first few weeks, and then stabilise at a plateau of activity thereafter. It is notable that, unlike previous studies [20], [22], we show a gradual decline in time spent running (Figure 2A) and distance run (Figure 2B) over the prolonged period of investigation, whereas average and maximum speed remain relatively consistent (Figure 2C–D). The days on which peak activity was recorded, and mean performance (±SD) after that day were as follows: Time spent running: peak day 29, mean during plateau phase 5.06±0.70 hours/day. Distance run: peak day 31, mean during plateau phase 15.07±1.78 km/day. Average and maximum speeds do not reach a peak until day 114 and 120 respectively, suggesting that these parameters continue to improve over time. Our results are comparable with previous investigations [20], [22], and suggest that C57BL/6 J OlaHsd substrain mice are suitable for the proposed study.

### Early read outs of motor dysfunction

**Figure 3:**
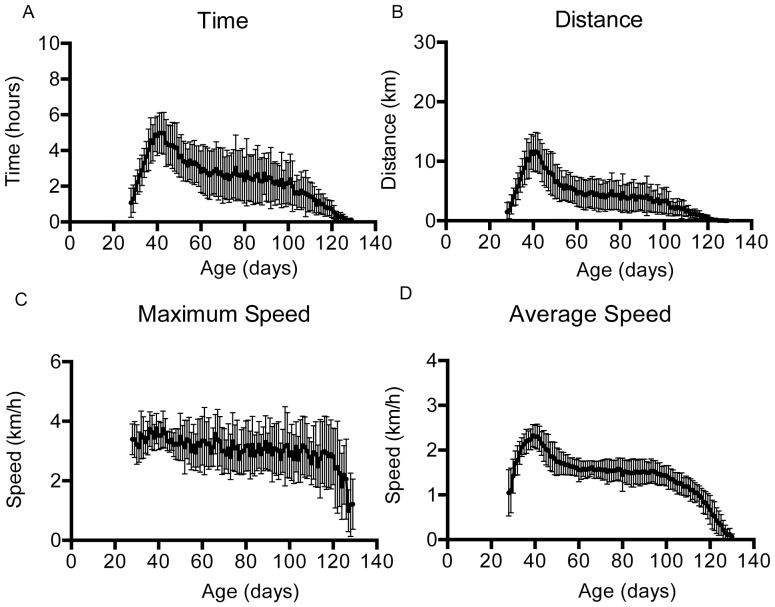
Running wheel activity of SOD1^G93A^ mice. Mean (± SD) time (A), distance (B), maximum speed (C) and average speed (D) were recorded in a group of 15 mice, starting from 25–31 days of age. Data are plotted from 28 days onwards, until the mice no longer used the running wheels.

To establish whether home cage running wheels allowed earlier detection of motor dysfunction, we investigated Fast Trac wheel-running in a cohort of 15 female SOD1^G93A^ mice. Because we previously showed a 20% reduction in rotarod performance in these mice at approximately 50 days of age, we placed mice in cages with running wheels as soon as possible following weaning, genotyping and transfer from SPF housing to the conventional facility. This varied between 25 and 31 days old. The graphs in Figure 3 show data collected from 28 days onwards, the point at which 50% of the cohort had been recruited. The time, distance and average speed of running wheel activity in the SOD1^G93A^ mice increases until approximately 40 days of age, when these parameters begin to decline until a plateau is reached at 60 days old. A further decrease in time, distance and average speed is observed between 100 and 130 days, at which point SOD1^G93A^ mice cease running. It is interesting to note that maximum running speed remains in the range of 2.8–3.5 km/h until mice are 120 days old.

**Figure 4:**
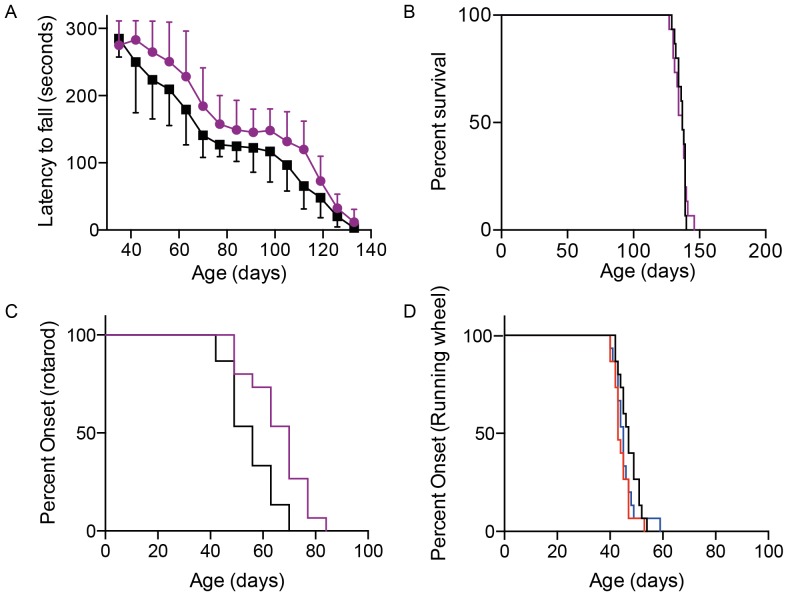
The effect of home cage running wheels on disease course in SOD1^G93A^ mice. Graphs showing rotarod (A), survival (B), onset determined by a 20% reduction in rotarod performance (C), and onset determined by a 20% decrease in running wheel activity (D) in littermate SOD1^G93A^ mice either with or without access to a home cage running wheel. In A–C mice with access to running wheels are shown in purple. In D onset determined by a 20% decline in the distance run is shown in red, time spent running is shown in blue and average speed is shown in black.

We previously showed that the time taken for a 20% reduction in latency to fall from the rotarod could be used as an easy to implement early readout of motor dysfunction in SOD1^G93A^ mice [11]. We calculated the mean age at which the distance run, time spent running and average speed of each mouse showed a 20% reduction. For distance run this was 44.3±3.3 days, for time spent running it was 45.5±4.5 days, and for average speed it was 47.1±3.8 days. Each of these is similar to the ages at which we previously reported a reduction of similar magnitude in rotarod performance (47.0 to 58.3 days) [11]. Interestingly, distance run (p<0.01) and time spent running (p<0.05) both detected a 20% decline in performance at an earlier stage than average speed (Kruskal-Wallis with Dunn's multiple comparisons test). In Figure 4D onset determined as a 20% reduction in running wheel activity is presented.

### Impact of wheel running on disease course

There are conflicting reports that voluntary exercise might influence the disease course in mutant SOD1 mouse models. To determine the effect of home cage voluntary running activity on disease course, in parallel to the above experiment we also housed transgenic female SOD1^G93A^ mice in identical cages, with non-rotating wheels. To control for litter effects, we used littermates of the SOD1^G93A^ mice on running wheels.

Mice from both cohorts (i.e. with and without running wheels) were tested weekly on an accelerating rotarod (Figure 4A). There was a clear improvement in rotarod performance in the mice with access to running wheels (Two-way ANOVA p<0.0001), although the same pattern of disease progression (early decline, plateau, and final decline in performance) was seen in both groups. This pattern correlates with the pathology that has been previously described in the SOD1^G93A^ model, with fast fatigable motor neuron endplates dying-back prior to symptom onset, fast non-fatigable endplates dying-back at onset, and slow endplates showing resistance [17]. The age at which we first observed a 20% reduction in rotarod performance was 55.1±9.1 days in non-running mice and 65.8±11.1 days in the running group. This was a significant difference (unpaired t test: p<0.01), suggesting a beneficial effect of wheel-running in this context. The data is also presented as a survival curve to allow direct comparison of onset between groups and between experiments (Figure 4C).

Mice were weighed weekly, and although there was no significant difference in peak body weight (15.9±0.7 g in non-running mice and 15.8±0.7 g in the running group), the age at which mice reached peak body weight was significantly later in the running-wheel group (64.4±22.0 days in non-running mice and 91.0±16.1 days in the running group. Mann-Whitney test: p<0.01).

Mice were scored weekly for disease onset defined by the age at which abnormal hindlimb splay and tremor were both observed. Using these criteria, onset was very similar in both groups: 77.6±7.1 in non-running mice and 78.6±10.1 in the running group (Mann-Whitney test: non-significant). Finally we determined the survival of both groups, defined as the point at which mice were unable to self-right within 10 seconds of being placed on the back. Using the Log-rank (Mantel-Cox) test there was no significant difference between running and non-running groups (Figure 4B).

### Running wheels provide a very reliable measure of disease onset

**Figure 5:**
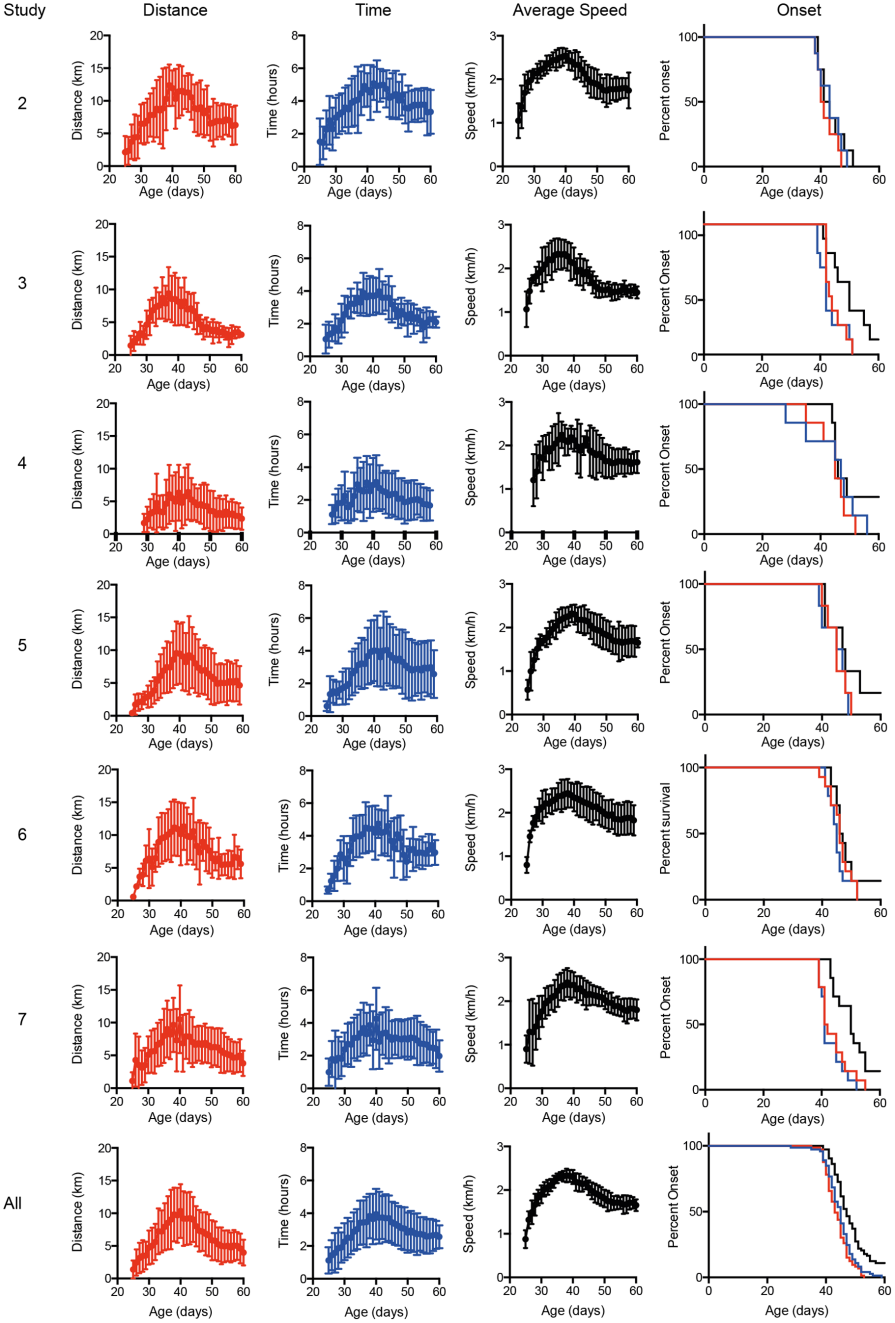
Reproducibility of running wheel average speed as an early readout of motor dysfunction. Data obtained from placebo control animals in six separate preclinical studies are shown (Studies 2–7). In addition, combined results from all seven studies are shown to illustrate the reproducibility of this methodology. Onset determined by a 20% decline in the distance run is shown red, time spent running is shown in blue and average speed is shown in black.

To determine whether home cage running wheel activity is a reliable readout of early motor dysfunction we analysed the data obtained from control groups used in 6 therapeutic studies run over an 18 month period (Table 1). The running wheel data for the 6 groups are shown in Figure 5. The results are highly reproducible between studies, and the average ages at which a 20% reduction in running wheel activity is observed are summarised in Table 1. Importantly each study shows a low coefficient of variation (CV), and analysis of all 72 mice investigated to date allows us to determine the CV to be as low as 9.0% for distance run and average speed. When considering the mean data obtained in each of the experiments, the “between study” CV is between 2.2 and 3.8% for the running wheel parameters. Using the mean and standard deviation of these 72 mice we have calculated that using treatment and control groups of 4 mice provides 80% power to detect a 10 day difference in the age at which a 20% reduction in distance run is observed (groups of 5 are powered to detect a 10 day difference in time spent running and average speed). This is a dramatic reduction in the number of animals required when compared to rotarod, which requires 14 mice per group for the same reduction compared to peak performance [11].

## Discussion

Animal models are essential for understanding disease mechanisms and developing treatments for ALS. We are fortunate that the SOD1^G93A^ mouse is a well-established model of ALS, with a widely accepted set of guidelines for use in preclinical studies [23], [24]. Despite this, the model can be refined to further enhance its usefulness. For instance, therapeutic studies using survival as an endpoint take 140 days to complete, which is significant in terms of cost and personnel. Also the welfare of SOD1^G93A^ mice is a serious concern, particularly in the latter stages of the disease. Thus it is desirable to refine the model to be able to use earlier endpoints.

It is now accepted that the earliest symptoms of ALS in human patients involve dying back of axons, and subsequent remodelling of neuromuscular junctions [15], [25]. SOD1^G93A^ mice mimic this early neuromuscular pathology faithfully, and we can use quantitative measurements of muscle weakness such as rotarod, grip-strength and wheel-running to follow the progressive deterioration of neuromuscular junctions during the disease course. It is rarely possible to measure the earliest stages of ALS in human patients using the same quantitative approach. However a prospective 3 year long study of presymptomatic SOD1 mutation carriers, two sisters with V148G SOD1 mutations both showed significant loss of abductor pollicis brevis motor units using MUNE, 6 months before alterations in grip strength were reported [26].

We previously showed that reductions in rotarod performance could be used as an early endpoint in SOD1^G93A^ mice [11]. However the rotarod has a number of disadvantages. The data are quite variable, which necessitates the use of 14 mice per group (therefore 28 in total for placebo and treatment groups). The test is artificial, involves forced activity and testing takes place during the daytime, when mice are normally sleeping. Rotarod testing is also time consuming for the investigator, especially if data are to be collected several times per week for each mouse. In the current investigation we compared the use of rotarod with home cage running wheels. In contrast to rotarod, this use of home cage running wheels is a voluntary activity, takes place during the night when mice are most active, and produces data every day for each mouse with minimal intervention and investigator involvement. We show that, like the rotarod, home cage running wheels allow us to detect an early reduction in motor performance. We are able to detect a 20% reduction in performance with reduced variability, when compared to rotarod. Therefore we are able to reduce the timescale of early readout studies to 60 days, and also reduce the number of mice used by >60%, to 4 per group (therefore 8 in total for placebo and treatment groups). Over this time course the mice show no classical signs of disease, obvious gait abnormalities or distress compared to non-transgenic littermates [11]. However, as we and others have demonstrated previously, this early decline in motor performance correlates with the loss of NMJ integrity in hindlimb muscles [11], [14]–[17]. As such it is a proxy for EMG recordings, which are the gold standard method for measuring motor units in mice and human ALS patients. However EMG is not suitable for daily testing in mice, so alternative approaches, such as running wheel activity, are a useful way of obtaining similar results.

The main goal of preclinical experiments is to increase lifespan, indeed this is the principal endpoint in human ALS clinical trials. Therefore we propose that early endpoints should not be seen as a complete replacement for preclinical survival studies. For individual therapeutic approaches we will still need to empirically determine whether changes in the early readouts will lead to changes at the later stages of disease. Survival studies are also important because the majority of clinical trials in ALS patients use survival at 12 or 18 months as the primary endpoint [1]. However, the small group sizes and rapid timescales permitted by use of running wheels do facilitate affordable large-scale therapeutic pre-screening in the SOD1^G93A^ mouse, as well as rapid validation of published positive effects in a second laboratory, one of the requirements for preclinical screening for ALS set out in the current guidelines [24]. Therapeutic agents that show significant improvements in running wheel activity could then be validated in a secondary survival study over a longer time-frame.

One interesting finding in this study was the effect of home cage running wheels on disease course in SOD1^G93A^ mice. The impact of exercise in ALS has been somewhat controversial. We found that voluntary running activity did not affect onset (defined by appearance of tremor and hindlimb splay defect) or survival in female SOD1^G93A^ mice. However, it did significantly modify the temporal nature of weight gain in the mice, and significantly improved the performance of SOD1^G93A^ mice on the rotarod. Previously it was reported that male, but not female C57BL/6 mice, given access to the same Fast Trac running wheels had a reduced weight after 5 weeks of exercise [20]. In keeping with this, after 5 weeks of exercise our female SOD1^G93A^ mice did not show a significant difference in weight compared to non-running SOD1^G93A^ littermates (data not shown). We are not able to explain the beneficial effects of running wheel activity on rotarod performance, beyond the possibility that this may reflect a training effect in the mice that exercise regularly. We previously showed that in female C57BL/6 mice home cage running wheels altered the transcriptome in both hindlimb muscles and spinal cord motor neurons [22]. It would be interesting to investigate whether any of the pathways identified by changes in gene expression, such as axon branching in motor neurons, or neovascularisation and myogenesis in muscle, are associated with the improvement in rotarod performance we report in exercised SOD1^G93A^ mice. However on balance, we are cautious about over-interpretation of this effect, since there was no improvement of overall survival in the mice with access to running wheels.

We also predict the usefulness of this approach in other settings, including new mouse models of ALS. New animal models of ALS are needed to understand disease mechanisms and therapeutics, and the discovery of new genes causing familial ALS mean that new models are likely to emerge in the next few years. However, despite the excitement in the ALS field surrounding TDP-43 mutations and the prevalence of TDP-43 as a pathological marker in ALS [27], there is no consensus opinion yet for a robust and useful animal model of TDP-43 related ALS that closely mimics the human disease [28].

In conclusion we present a method for very rapid, sensitive, and reliable measurement of motor dysfunction in SOD1^G93A^ mice, which offers significant advantages for refinement and reduction of animal use in ALS preclinical testing.
